# Two Cases of Epidermal Growth Factor Receptor L861R Mutation‐Positive Lung Adenocarcinoma Treated With Osimertinib and Afatinib

**DOI:** 10.1002/cnr2.70420

**Published:** 2025-12-05

**Authors:** Kei Kagawa, Takeshi Masuda, Kiyofumi Shimoji, Kakuhiro Yamaguchi, Shinjiro Sakamoto, Yasushi Horimasu, Taku Nakashima, Hiroshi Iwamoto, Hironobu Hamada, Noboru Hattori

**Affiliations:** ^1^ Department of Respiratory Medicine Hiroshima University Hospital Hiroshima Japan; ^2^ Department of Respiratory Medicine Chugoku Rosai Hospital Kure Japan; ^3^ Department of Molecular and Internal Medicine, Graduate School of Biomedical and Health Sciences Hiroshima University Hiroshima Japan; ^4^ Department of Physical Analysis and Therapeutic Sciences Hiroshima University Hiroshima Japan

**Keywords:** afatinib, epidermal growth factor receptor mutation, L861R, lung adenocarcinoma, osimertinib

## Abstract

**Background:**

Epidermal growth factor receptor (EGFR) mutations are detected in approximately 40%–50% of patients with lung adenocarcinoma in Asian populations and in around 10% of patients in Western populations. Among these, the EGFR L861R mutation is uncommon and undetectable using conventional polymerase chain reaction methods. Recently, clinically available next‐generation sequencing (NGS) has been used to detect L861R mutations, potentially increasing the number of identified cases of L861R‐positive non‐small cell lung cancer. Herein, we present two cases of EGFR L861R‐mutated lung adenocarcinoma treated with afatinib and osimertinib to evaluate the clinical efficacy and tolerability of these targeted therapies.

**Case:**

Case 1: A 63‐year‐old man with lung adenocarcinoma harboring an EGFR L861R mutation (cStage IVB) received afatinib 40 mg/day. Computer tomography (CT) on Day 49 showed shrinkage of the primary tumor, accompanied by a decrease in tumor markers. The afatinib dose was reduced to 30 mg/day due to Grade 1 diarrhea and an acneiform rash. Ground‐glass opacities were subsequently observed around the tumor. Although pneumonitis was initially suspected, the findings were subsequently considered consistent with carcinomatous lymphangitis. Consequently, afatinib was discontinued on Day 56. Case 2: An 83‐year‐old man with lung adenocarcinoma harboring an EGFR L861R mutation (cStage IVB) received osimertinib 80 mg/day. Afatinib was avoided due to concerns regarding tolerability in the elderly. CT imaging on Day 14 showed tumor shrinkage with reduced attenuation and decreased levels of tumor markers. However, osimertinib was discontinued on Day 104 due to a Grade 3 skin rash. The response to osimertinib was evaluated as stable disease. Following treatment discontinuation, the disease progressed to multiple brain metastases, and supportive care was initiated.

**Conclusion:**

Here, we present the first case showing the anti‐tumor efficacy of afatinib and the second case showing the anti‐tumor efficacy of osimertinib in a patient with EGFR L861R‐positive lung adenocarcinoma. Despite their short treatment durations, afatinib and osimertinib may have potential clinical activity in patients with EGFR L861R‐positive lung cancer.

## 1 Introduction

Among epidermal growth factor receptor (EGFR) mutations, exon19 deletion and L858R point mutation, referred to as major or classical mutations, account for 85% of lung adenocarcinoma [1]. In addition, many minor mutations have been reported. The incidence of G719X and L861Q has been reported to be 3.1% and 0.9%, respectively. However, the incidence of the L861R mutation remains unknown [2, 3]. This is because conventional polymerase chain reaction methods cannot detect L861R mutations. Recently, advances in next‐generation sequencing, including platforms such as the Oncomine Dx Target Test Multi‐CDx System (Oncomine) and Lung Cancer Compact Panel Dx Multi‐Companion Diagnostic System, have enabled the detection of the L861R mutation. Thus, the number of L861R‐positive identified patients may increase.

Current international guidelines primarily focus on the classical EGFR mutations and provide specific treatment recommendations for uncommon mutations such as EGFR S768I, L861Q, and G719X. In patients with these uncommon EGFR mutations, a phase 3 study showed that the median progression‐free survival (PFS) was significantly longer with afatinib treatment compared with chemotherapy [4]. Moreover, phase 2 studies showed that osimertinib had a PFS of 9.4 and 8.2 months [5, 6]. Based on these results, afatinib or osimertinib is preferred in international guidelines for patients with minor EGFR mutations, such as EGFR S768I, L861Q and G719X [7, 8, 9]. To date, only one case report has described the efficacy of osimertinib in a patient with an EGFR L861R mutation [10], and there are no specific treatment recommendations for EGFR L861R in the current international guidelines.

Here, we report the first case demonstrating the anti‐tumor efficacy of afatinib and the second case demonstrating the efficacy of osimertinib.

## 2 Case

### 2.1 Case 1

**FIGURE 2:**
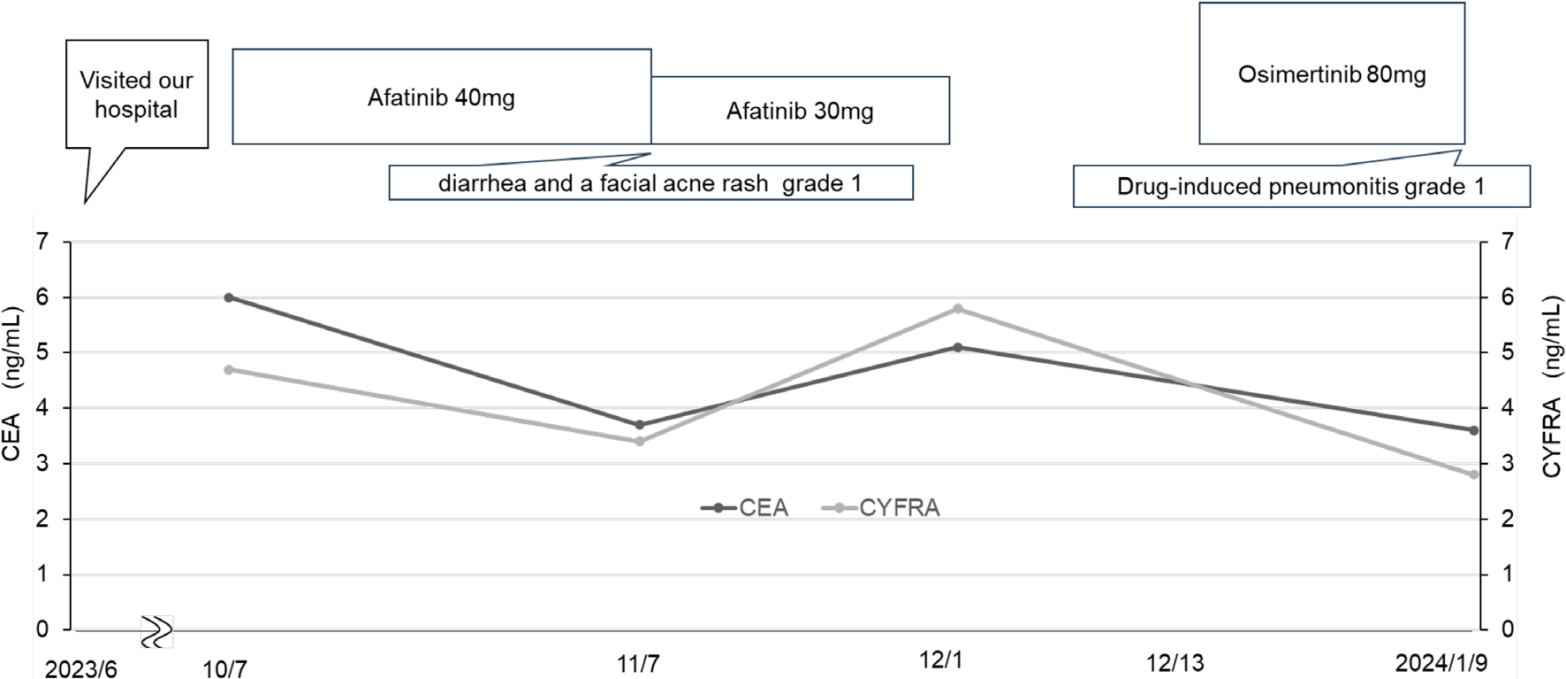
Clinical course since the start of treatment. CEA, carcinoembryonic antigen; CYFRA, cytokeratin 19 fragment.

A 63‐year‐old man with no history of smoking and an Eastern Cooperative Oncology Group (ECOG) performance status of 0 underwent routine chest radiography during an annual health checkup in June 2023, which revealed an abnormal pulmonary shadow. He later presented to Hiroshima University Hospital for further evaluation. He had a history of hyperuricemia and was receiving febuxostat. Laboratory tests revealed elevated tumor markers, including carcinoembryonic antigen (CEA) (5.5 ng/mL) and cytokeratin 19 fragment (CYFRA 21‐1) (5.1 ng/mL) (Table 1). Positron emission tomography/computed tomography (PET/CT) showed metastatic lymph nodes, lung, and bone tumors. The patient was diagnosed with clinical T4N3M1c stage IVB lung adenocarcinoma in June 2023 (Figure 1A). The Oncomine Dx Target Test performed on the transbronchial biopsy tissues identified the EGFR L861R mutation. Afatinib was initiated at 40 mg/day (Figure 2). By Day 32, tumor marker levels had decreased; however, due to Grade 1 diarrhea and facial acneiform rash, the dose of afatinib was reduced from 40 to 30 mg. On Day 49, CT revealed that the primary lung tumor had shrunk (Figure 1B), but also revealed ground‐glass opacities (GGO) and consolidation around the tumor, suggesting afatinib‐induced pneumonitis or carcinomatous lymphangitis (Figure 1C). On Day 56, CEA levels increased. Furthermore, pulmonary opacities did not change after the cessation of afatinib treatment from Days 56 to 69. An increase in CEA levels indicates the proliferation of adenocarcinoma cells. If the opacities were attributable to afatinib‐induced pneumonitis, they would have improved after the discontinuation of afatinib; however, the findings did not improve despite cessation of the drug. Although a tissue diagnosis using bronchoscopy was not performed, and the diagnosis remained provisional, we considered that the opacities were due to carcinomatous lymphangitis. Owing to toxicity, afatinib re‐escalation was not feasible. Therefore, we switched to osimertinib (80 mg/day), which was expected to provide clinical efficacy and could be administered at the full recommended dose. Tumor marker levels decreased, and CT showed that the size of the primary tumor remained unchanged (Figure 1D). However, CT revealed the development of new GGOs (Figure 1D,E). Osimertinib was discontinued on Day 112. Subsequently, the opacities resolved, and the patient was diagnosed with afatinib‐ and osimertinib‐induced pneumonitis. Pneumonitis was assessed as CTCAE Grade 1 [11], and the patient remained asymptomatic. Corticosteroid therapy was not initiated; instead, treatment was withdrawn, and the patient was managed with close observation alone.

**TABLE 1:** Laboratory findings at the first visit.

Hematology	
WBC	5880/μL
Ne	65.9%
Ly	23.2%
Mo	7.7%
Eo	2.6%
Ba	0.2%
RBC	5.22 × 10^6^/μL
Hb	15.1 g/dL
Ht	47.6%
PLT	27 × 10^4^/μL
Biochemistry	
TP	7.2 g/dL
ALB	3.8 g/dL
T‐Bil	0.4 mg/dL
AST	24 U/L
ALT	16 U/L
LDH	176 U/L
BUN	16.8 mg/dL
Cre	1.07 mg/dL
UA	5.6 mg/dL
Na	138 mEq/L
K	4.5 mEq/L
Cl	102 mEq/L
Ca	9.1 mEq/L
Glucose	90 mg/dL
Immunology	
CRP	0.31 mg/dL
KL‐6	2858 U/mL
Tumor marker	
CEA	5.5 ng/mL
CYFRA21‐1	5.1 ng/mL

Abbreviations: Alb: albumin, ALT: alanine transaminase, AST: aspartate aminotransferase, Ba: basophil, BUN: blood urea nitrogen, Ca: calcium, CEA: Carcinoembryonic Antigen, Cl: chloride, Cre: creatinine, CRP: C‐reactive protein, CYFRA21‐1: Cytokeratin 19 fragment, Eo: eosinophil, Hb: hemoglobin, Ht: hematocrit, K: potassium, KL‐6: Krebs von den Lungen‐6, LDH: lactate dehydrogenase, Ly: lymphocyte, Mo: monocyte, Na: sodium, Ne: neutrophil, PLT: platelet, RBC: red blood cell, T‐Bil: total bilirubin, TP: total protein, UA: uric acid, WBC: white blood cell.

**454544FIGURE 1:**
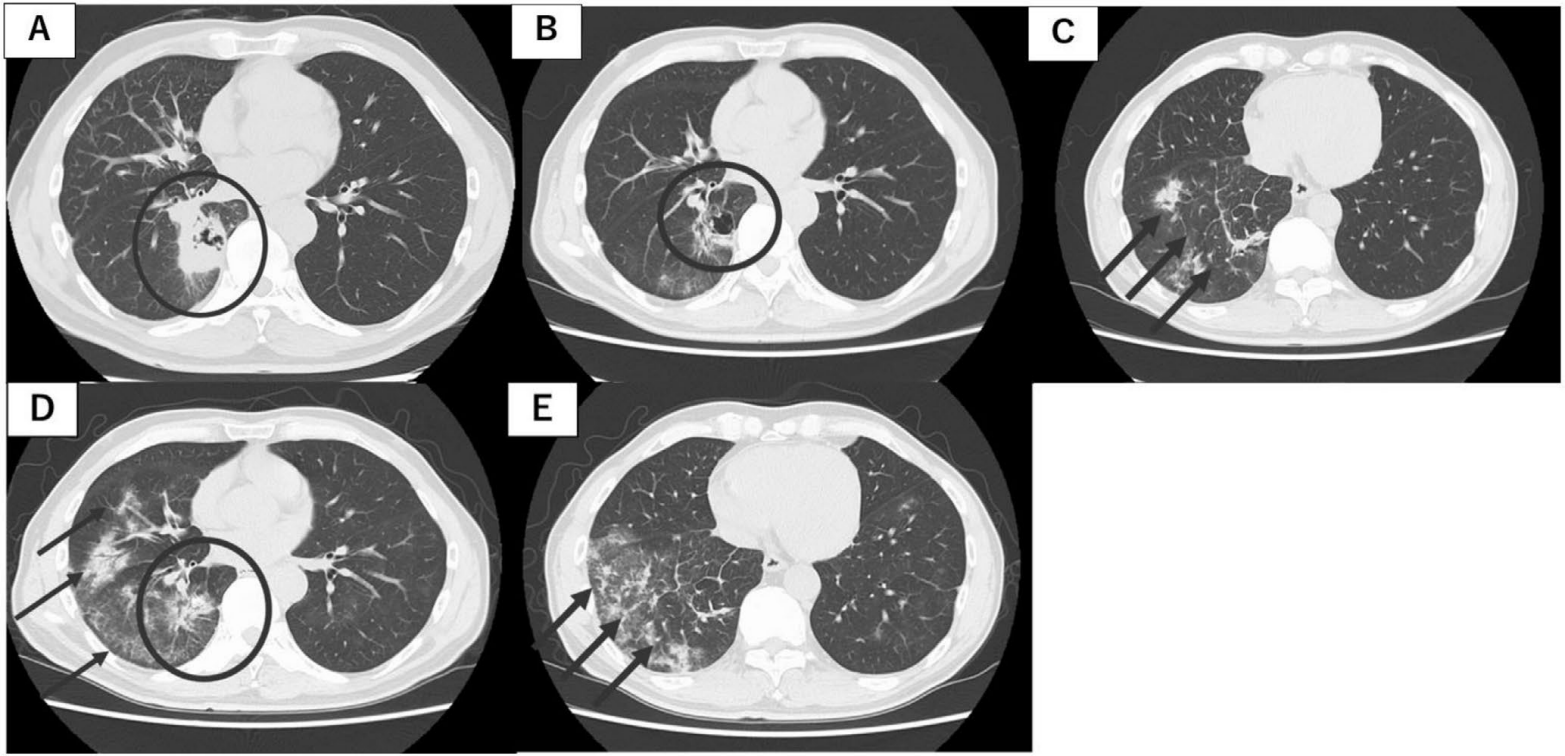
(A) Computed tomography (CT) showed a primary tumor in the S6 of the right lung (circle). (B) CT performed on Day 49. The primary lung tumor had shrunk (circle). (C) New ground‐glass opacity and consolidation appeared around the tumor (arrow). (D) CT showed the size of the primary tumor remained unchanged (circle). (D, E) ground‐glass opacity shadow developed (arrow).

Afatinib induced a reduction in the size of the primary lung tumor, and osimertinib maintained stable disease [12], with treatment durations of 56 and 43 days, respectively. After the discontinuation of osimertinib, the patient received carboplatin (CBDCA) plus pemetrexed plus bevacizumab, followed by osimertinib rechallenge and CBDCA plus nab‐paclitaxel. As of September 2025, the patient remains alive with an overall survival of 27 months.

### 2.2 Case 2

**FIGURE 3:**
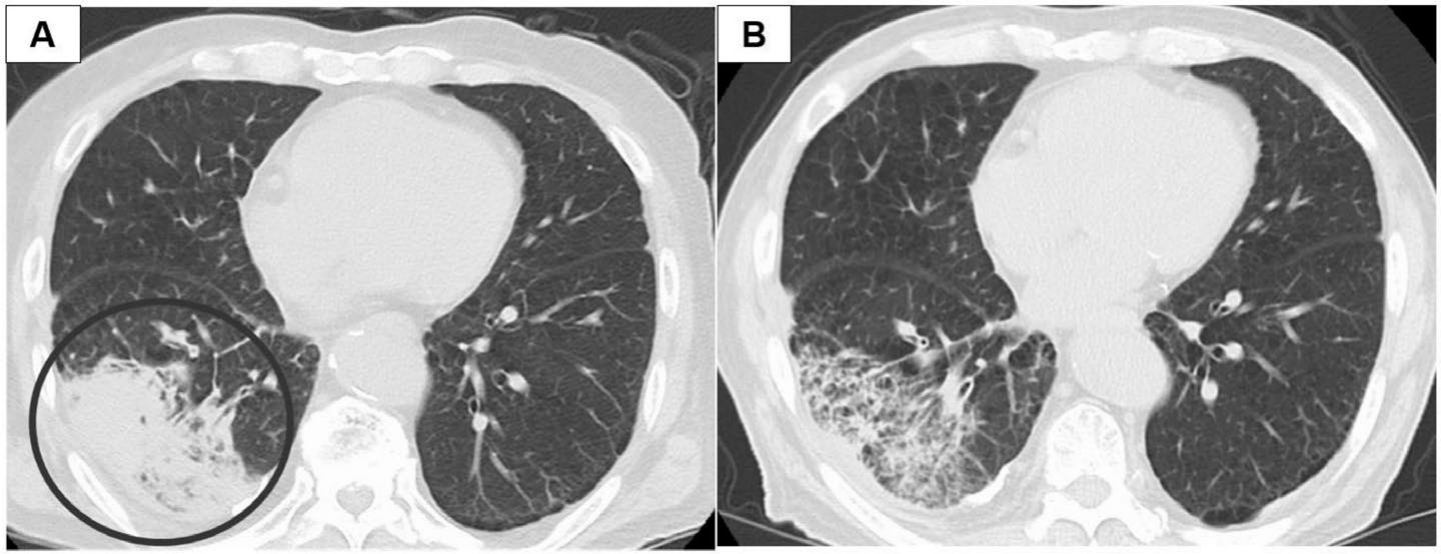
(A) Computed tomography showed a primary tumor in the S9 of the right lung (circle). (B) Computed tomography showed that the tumor had shrunk and its attenuation had decreased.

**FIGURE 4:**
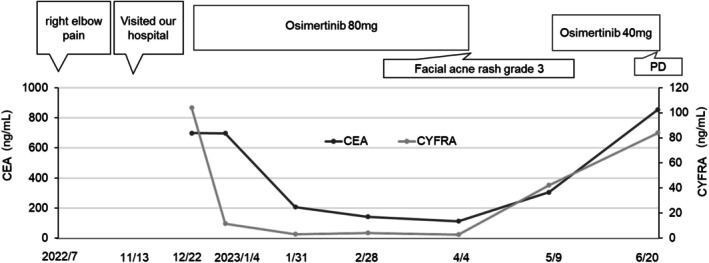
Clinical course since the start of treatment. PD, progressive disease; CEA, carcinoembryonic antigen; CYFRA, cytokeratin 19 fragment.

An 83‐year‐old man complained of right elbow pain in July 2022, and was referred to the Hiroshima University Hospital in November 2022 for further management. He was a former smoker with a performance status of 0. His medical history included hypertension, diabetes, and hyperlipidemia for which he was taking olmesartan, amlodipine, sitagliptin, and rosuvastatin. Laboratory tests revealed leukocytosis (white blood cell count, 10 960/μL), elevated lactate dehydrogenase (401 U/L), and markedly increased tumor markers, including carcinoembryonic antigen (CEA) (525.1 ng/mL) and cytokeratin 19 fragment (CYFRA 21‐1) (46.5 ng/mL) (Table 2). PET/CT revealed metastatic adrenal glands, liver, and bone tumors. MRI showed no evidence of brain metastases. The patient was diagnosed with cT4N2M1c stage IVB lung adenocarcinoma (Figure 3A). The Oncomine Dx Target Test performed on a right radial head biopsy sample identified an EGFR L861R mutation. Given concerns about tolerability and adverse reactions—particularly the potential for skin rash and diarrhea due to afatinib treatment, osimertinib 80 mg/day was initiated (Figure 4). CT imaging on Day 14 showed that the tumor had shrunk and its attenuation had decreased (Figure 3B), with decreased tumor markers. Tumor marker levels remained low; however, osimertinib was discontinued on Day 104 due to a Grade 3 skin rash [11]. Considering that the patient had benefited from osimertinib, continuation of treatment at a reduced dose was considered a reasonable approach. Alternative therapies such as carboplatin plus pemetrexed, docetaxel, or afatinib were considered; however, given the patient's advanced age (83 years), the tolerability of these regimens was expected to be poor. Therefore, osimertinib was reintroduced at a reduced dose of 40 mg after the rash improved to Grade 1. Despite this, the tumor marker levels continued to rise. Six months after starting osimertinib, CT revealed progression of the primary tumor and the emergence of multiple brain metastases. The PS dropped to 4, and best supportive care was initiated. The patient remained alive until July, 2023. The patient was then transferred to a palliative care hospital. Follow‐up was available up to this point, with a total observation period of approximately 6.5 months. The efficacy of osimertinib was evaluated as stable disease [12], with a treatment duration of 3.5 months.

**TABLE 2:** Laboratory findings at the first visit.

Hematology	
WBC	10 960/μL
Ne	58.6%
Ly	32.0%
Mo	7.3%
Eo	1.7%
Ba	0.4%
RBC	4.76 × 10^6^/μL
Hb	13.7 g/dL
Ht	43%
PLT	30.4 × 10^4^/μL
Biochemistry	
TP	7.6 g/dL
ALB	3.9 g/dL
T‐Bil	0.7 mg/dL
AST	28 U/L
ALT	15 U/L
LDH	401 U/L
BUN	17.9 mg/dL
Cre	0.88 mg/dL
UA	5.7 mg/dL
Na	139 mEq/L
K	4.2 mEq/L
Cl	104 mEq/L
Ca	9.5 mEq/L
Glucose	123 mg/dL
Immunology	
CRP	0.45 mg/dL
KL‐6	602 U/mL
Tumor marker	
CEA	525.1 ng/mL
CYFRA21‐1	46.5 ng/mL

Abbreviations: Alb: albumin, ALT: alanine transaminase, AST: aspartate aminotransferase, Ba: basophil, BUN: blood urea nitrogen, Ca: calcium, CEA: Carcinoembryonic Antigen, Cl: chloride, Cre: creatinine, CRP: C‐reactive protein, CYFRA21‐1: Cytokeratin 19 fragment, Eo: eosinophil, Hb: hemoglobin, Ht: hematocrit, K: potassium, KL‐6: Krebs von den Lungen‐6, LDH: lactate dehydrogenase, Ly: lymphocyte, Mo: monocyte, Na: sodium, Ne: neutrophil, PLT: platelet, RBC: red blood cell, T‐Bil: total bilirubin, TP: total protein, UA: uric acid, WBC: white blood cell.

## 3 Discussion

Here, we present the first case demonstrating the anti‐tumor efficacy of afatinib in a patient with EGFR L861R‐positive lung adenocarcinoma. Despite the short duration of administration of these drugs owing to adverse events, the tumor size decreased after treatment. This observation is the most significant finding of this study. A previous study showed that a patient with EGFR L861R‐positive lung adenocarcinoma treated with afatinib and bevacizumab as second‐line treatment had a PFS of 2 months. Notably, this patient had an EGFR L858R mutation initially and was treated with osimertinib as a first‐line treatment for 10 months; next‐generation sequencing detected the EGFR mutation L861R in the plasma [13]. Thus, the treatment lines and samples for EGFR mutation testing were different between cases and may account for the difference in outcomes. Another case of lung adenocarcinoma harboring EGFR S720F and L861R mutations has been reported [14]. The patient was treated with afatinib, and PFS was 16.0 months. In contrast, we demonstrated the efficacy of afatinib in patients with a single EGFR L861R mutation, but not in those with compound mutations such as S720F and L861R, which might influence treatment response. Regarding osimertinib, only one prior report has documented its efficacy on an EGFR L861R‐positive case, with PFS of 16 months [10]. Our patient, harboring the EGFR L861R mutation, treated with osimertinib, is the second reported case. Although osimertinib and afatinib have anti‐tumor efficacy against EGFR L861R‐positive lung adenocarcinoma, we should investigate the efficacy of EGFR‐TKIs in EGFR L861 mutation‐positive cases using a large number of cases. Given the rarity of EGFR L861R mutations, treatment decisions must be individualized, carefully considering both the efficacy and tolerability, particularly in elderly patients. In this context, case reports provide valuable clinical insights and contribute to building an evidence base that may guide therapeutic strategies for patients with uncommon EGFR mutations. Further accumulation of such case reports is important to establish optimal treatment approaches for patients with EGFR L861R‐positive lung cancer.

Preclinical data from Kohsaka et al. showed the minimum concentrations at which anti‐tumor efficacy could be achieved against EGFR L861R mutation‐positive cells [15]. The minimum concentrations of afatinib and osimertinib were 0.001 and 0.01 μM, respectively, indicating that afatinib has a higher anti‐tumor efficacy. Therefore, afatinib was used as the first‐line treatment in the first case. In the second case, osimertinib resulted in a PFS of 3.5 months; however, the patient was not treated long‐term because of toxicity. Further evaluation of the efficacy of both drugs is necessary in a larger cohort. Although there were limitations in extrapolating preclinical data to clinical outcomes, we used in vitro experimental data to guide the selection of EGFR‐TKIs.

Focusing on toxicity in elderly patients with EGFR‐mutated NSCLC, previous studies have shown that the incidence of Grade 3 skin rash, diarrhea, and pneumonitis was 14%, 13%, and 2.9%, respectively, with afatinib [16]. In contrast, the incidence of Grade 3 skin rash, diarrhea, and pneumonitis was only 5.0%, 0%, and 2.5%, respectively, with osimertinib [17], suggesting that osimertinib is superior in terms of toxicity. Therefore, osimertinib is a suitable treatment option for elderly patients harboring the EGFR L861R mutation.

The main limitation of this study is the lack of long‐term follow‐up, which makes it difficult to draw definitive conclusions regarding the durability of treatment efficacy. In addition, another limitation is that we were unable to clearly demonstrate the disease response to afatinib or osimertinib with imaging findings. Further prospective studies with larger patient cohorts and extended follow‐ups are warranted to establish optimal therapeutic strategies for EGFR L861R–positive lung cancer.

## 4 Conclusion

Here, we present the first case showing the anti‐tumor efficacy of afatinib and the second case showing the anti‐tumor efficacy of osimertinib, respectively, in patients with EGFR L861R‐positive lung adenocarcinoma. Despite their short treatment durations, afatinib and osimertinib may have potential clinical activity in patients with EGFR L861R‐positive lung cancer.

When performing driver mutation testing in patients with lung adenocarcinoma, it is preferable to use next‐generation sequencing (NGS), which can detect a broad spectrum of mutations, including EGFR L861R, rather than a single‐plex EGFR assay. If EGFR L861R is detected, afatinib or osimertinib should be considered as the recommended treatment options for patients with EGFR L861R–positive lung cancer. Osimertinib may be better tolerated by elderly patients.

## Author Contributions

Conceptualization: Kei Kagawa and Takeshi Masuda. Data curation: Kei Kagawa, Takeshi Masuda. Supervision: Noboru Hattori. Writing the original draft: Kei Kagawa and Takeshi Masuda. Writing – review and editing: Kiyofumi Shimoji, Kakuhiro Yamaguchi, Shinjiro Sakamoto, Yasushi Horimasu, Taku Nakashima, Hiroshi Iwamoto, Hironobu Hamada, and Noboru Hattori.

## Funding

The authors have nothing to report.

## Ethics Statement

Ethical approval was not required for this study in accordance with local or national guidelines.

## Consent

The patients and their family gave informed consent to publish this report.

## Conflicts of Interest

Takeshi Masuda received speaker honoraria from Daiichi‐Sankyo Co. Ltd., Taiho Pharmaceutical Co. Ltd., Nippon Boehringer Ingelheim Co. Ltd., Kyowa Kirin Co. Ltd., Eli Lilly Japan K. K., Ono Pharmaceutical Co. Ltd., Otsuka Pharmaceutical Co. Ltd., Chugai Pharmaceutical Co. Ltd., and AstraZeneca K. K., outside the submitted work. Kakuhiro Yamaguchi received speaker honoraria from Ono Pharmaceutical Co. Ltd., Chugai Pharmaceutical Co. Ltd., and Bristol‐Myers Squibb Co. Ltd., outside the submitted work. Shinjiro Sakamoto received speaker honoraria from Nippon Boehringer Ingelheim Co. Ltd., Ono Pharmaceutical Co. Ltd., MSD K.K., Chugai Pharmaceutical Co. Ltd., AstraZeneca K.K., and Kyowa Kirin Co. Ltd., outside the submitted work. Yasushi Horimasu received a speaker honorarium from AstraZeneca K.K., Nippon Boehringer Ingelheim Co. Ltd., and Asahi Kasei Pharma Co. Ltd. and received a manuscript fee from Nankodo Co. Ltd. and Japan Medical Journal Co. Ltd., outside the submitted work. Taku Nakashima received a speaker honorarium from AstraZeneca K.K. and research funding from Boehringer Ingelheim Co. Ltd., outside the submitted work. Noboru Hattori received a speaker honorarium from Nippon Boehringer Ingelheim Co. Ltd., Chugai Pharmaceutical Co. Ltd., and AstraZeneca K.K., and donations from Taiho Pharmaceutical Co. Ltd., Kyowa Kirin Co. Ltd., Nippon Boehringer Ingelheim Co. Ltd., and Chugai Pharmaceutical Co. Ltd., outside the submitted work. The supporting source and financial relationships had no involvement related to the present study.

## Data Availability

The data supporting this case report are not publicly available due to patient privacy and ethical restrictions. All relevant information is included within the article.
